# The disc damage likelihood scale: Diagnostic accuracy and correlations with cup-to-disc ratio, structural tests and standard automated perimetry

**DOI:** 10.1371/journal.pone.0181428

**Published:** 2017-07-20

**Authors:** Andrea C. Kara-José, Luiz Alberto S. Melo, Bruno L. B. Esporcatte, Angelica T. N. H. Endo, Mauro Toledo Leite, Ivan Maynart Tavares

**Affiliations:** Glaucoma Division, Department of Ophthalmology and Visual Sciences, Escola Paulista de Medicina, Universidade Federal de Sao Paulo, Sao Paulo, SP, Brazil; Oregon Health and Science University, UNITED STATES

## Abstract

Our objective was to compare the diagnostic accuracies of and to determine the correlations between the disc damage likelihood scale (DDLS) and anatomical and functional tests used for glaucoma detection. A total of 54 healthy subjects (54 eyes) and 47 primary open-angle glaucoma patients (47 eyes) were included in this cross-sectional observational study. DDLS scores and cup-to-disc (C/D) ratios were evaluated. Subjects underwent standard automated perimetry (SAP), optic disc and retinal nerve fiber layer (RNFL) imaging with time and spectral-domain optical coherence tomography (TD and SD-OCT), Heidelberg Retina Tomograph (HRT II), and scanning laser polarimetry (GDx-VCC). Areas under the receiver operating characteristic curves (AROCs) for DDLS and diagnostic tests parameters were calculated. DDLS correlations (Spearman's rank) among these parameters were analyzed. Fifty-four eyes were healthy and 47 had glaucoma, including 16 preperimetric glaucoma. DDLS, vertical and horizontal C/D ratios had the largest AROCs (0.92, 0.94 and 0.91, respectively). DDLS diagnostic accuracy was better than the accuracies of HRT II parameters, TD and SD-OCT RNFL thicknesses, and SAP mean deviation (MD) index. There were no significant differences between the accuracies of the DDLS and the C/D ratios, TD-OCT *vertical* (0.89) and *horizontal* (0.86) *C/D ratios*, TD-OCT *C/D area ratio* (0.89), and GDx-VCC *NFI* (0.81). DDLS showed significant strong correlations with vertical (*r* = 0.79) and horizontal (0.74) C/D ratios, and with the parameters *vertical C/D ratio* and *C/D area ratio* from HRT II (both 0.77) and TD-OCT (0.75 and 0.72, respectively). DDLS had significant moderate correlations with most of the other structural measurements and SAP MD. The optic disc clinical evaluation with DDLS system and C/D ratio demonstrated excellent accuracy in distinguishing glaucomatous from healthy eyes. DDLS had moderate to strong correlations with most structural and functional parameters. These findings stress the importance of optic disc clinical examination to detect glaucoma in a clinical scenario.

## Introduction

Glaucoma is an optic neuropathy characterized by progressive degeneration of retinal ganglion cells (RGC), with resultant structural changes to the optic nerve head (ONH), retinal nerve fiber layer (RNFL), and specific visual field loss.[1, 2] The RGC are central nervous system neurons that have their cell bodies in the inner retina and axons in the optic nerve.[3] Degeneration of these nerves results in neuroretinal rim thinning and consequent *cupping*, a characteristic appearance of the optic disc, and in visual loss.[1, 3]

There are different methods for ONH clinical classification and the cup-to-disc (C/D) ratio is the most commonly used. Clinical evaluation of C/D ratio is readily available; however, its assessment of the ONH is subjective, has fair-to-moderate inter- and intra-observer agreement, and does not consider optic disc size nor the cup position.[4–9] On the other hand, the disc damage likelihood scale (DDLS), developed by Spaeth et al.,[8, 10] aims to minimize these issues by taking into account the rim configuration, adjusted for the disc size, to estimate the optic disc health.[8, 10, 11] This quantitative 10 stage system grades the optic disc according to amount of damage based on the narrowest width of the rim and on the vertical disc diameter, therefore reducing the influence of disc size on ONH evaluation.[8, 10] The DDLS has low cost, good inter- and intra-observer agreement and good accuracy for glaucoma optic neuropathy diagnosis. In addition, this system correlates well with C/D ratio, standard automated perimetry, and with Heidelberg Retina Tomograph (HRT), Cirrus and Stratus optical coherence tomography (OCT) measurements; it has limitations though, such as a learning curve.[7, 8, 10, 12–21]

In the last few years, computerized imaging methods, such as OCT, confocal scanning laser ophthalmoscope (CSLO; HRT), and scanning laser polarimetry (SLP; GDx) have been thoroughly studied to offer an objective and automated quantitative structural evaluation of the ONH and RNFL, thus improving glaucoma diagnosis accuracy.[22–25] Nevertheless, these technologies have some limitations: they offer no qualitative disc evaluation, they are expensive, and they keep evolving, which limits glaucoma follow-up.[8]

The purpose of our study was to evaluate the ability of the DDLS system to detect glaucoma and to compare its diagnostic accuracy to C/D ratio, standard automated perimetry (SAP) mean deviation (MD) index, and to parameters of time and spectral-domain OCT (TD and SD-OCT), SLP (GDx-VCC), and CSLO (HRT II). Also, DDLS correlations among these parameters were analyzed.

## Materials and methods

Healthy and primary open-angle glaucoma (POAG) patients were included in this cross-sectional observational study. Glaucoma patients were recruited from the Glaucoma and General Clinic at the Ophthalmology Department of the Federal University of Sao Paulo, Brazil. Healthy subjects were recruited from the General clinic, hospital staff, and patient's relatives and companions. The study protocol and informed consent were approved by the Institutional Ethics Committee of the Federal University of São Paulo (1438/05) and followed the tenets of the Declaration of Helsinki. All participants provided their written informed consent to participate in this study. The data were collected after the protocol approval.

All subjects underwent a comprehensive ophthalmologic examination by a glaucoma specialist. The examination included review of medical history, subjective refraction, best-corrected visual acuity, slit-lamp biomicroscopy, Goldmann applanation tonometry, gonioscopy and dilated fundus biomicroscopy with 78-diopter (D) lens as well as dilated stereoscopic optic disc photography, SAP, SLP, CSLO, TD and SD-OCT. All of these exams were obtained within a 6-month period. Glaucomatous eyes were POAG cases, with intraocular pressure under the target level on hypotensive medications, probably with no significant progression during this period of time.

The inclusion criteria were: 1) best-corrected visual acuity of 20/40 or better for healthy participants, and 20/80 or better for glaucoma patients; 2) spherical refraction within ± 5.0D; and cylinder correction within ± 3.0 D; and 3) open angles on gonioscopy. The exclusion criteria were: 1) presence of ocular media opacities that interfere with the exams; 2) anterior segment abnormalities (except alterations caused by uncomplicated glaucoma or cataract surgery); 3) presence of other intraocular or neurological diseases affecting the RNFL, optic disc, or visual field; and 4) abnormal appearance of ONH, such as tilted disc, non-glaucomatous disc damage, or extensive peripapillary atrophy. Other exclusion criteria included history of intraocular trauma and surgery (except uncomplicated cataract or glaucoma surgery at least six months prior to examinations), subjects < 40 years of age and with inability to perform reliable perimetry (defined as rates of false positive < 15%, fixation loss < 20%, and false negative < 33%, with no visual field artifacts).

Glaucomatous eyes were defined as those with glaucomatous optic neuropathy (localized or diffuse rim or RNFL thinning) as assessed by masked examination of optic disc stereophotographs, regardless of intraocular pressure (IOP). Glaucoma was classified as preperimetric if the patient had normal SAP and as perimetric if the patient had two consecutive reliable and repeatable abnormal SAP results. An abnormal SAP result was defined as presence of one of the following criteria: 1) glaucoma hemifield test (GHT) outside normal limits; 2) pattern standard deviation (PSD) outside the 95% confidence limits; or 3) a pattern deviation plot with a cluster of > 3 non-edge points at p < 5%, with at least one point at p <1% in two consecutive fields. Healthy eyes had normal-appearing ONH and RNFL, IOP < 21 mmHg (with no history of elevated IOP), and normal SAP results.

**Fig 1:**
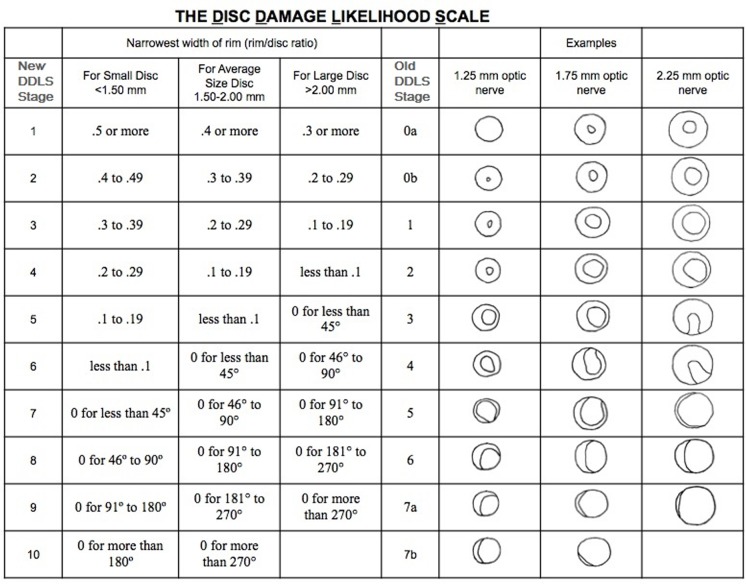
The Disc Damage Likelihood Scale (DDLS) nomogram. Figure adapted from the original, [11] and reprinted with permission from Elsevier.

The DDLS scores were calculated by a glaucoma specialist, at a slit lamp, using a 78 D noncontact fundus aspheric lens (Volk, Mentor, Ohio, USA). The optic disc size was measured using the adjustable slit lamp's slit beam and the fundus lens with a correction factor of 1.1.[8, 26] The DDLS staging is based on measuring the vertical optic disc diameter and assessing the narrowest width of the neuroretinal rim (rim/disc ratio) in any position; alternatively, in the case of rim absence, the circumferential extension of rim absence is measured in degrees (Fig 1).[8, 11] ONH is then staged based on the DDLS nomogram (Fig 1). Our study used the most recent version of this system, which stages the ONH from 1 to 10 (Fig 1).[8] DDLS scores > 5 indicate ONH glaucomatous damage (Fig 1). [8, 11]

### Instrumentation

SAP was performed with the Humphrey Field Analyzer (HFA II; Carl Zeiss Meditec, Dublin, CA, USA) Swedish interactive threshold algorithm standard 24–2 test.

Good quality color and red-free optic disc and RNFL photographs were taken with the Visucam Pro NM (Carl Zeiss Meditec, Dublin, CA, USA). The stereoscopic images were evaluated with a stereoscopic viewer (Screen-Vu stereoscope; PS Manufacturing, Portland, Oregon, USA). Two glaucoma specialists blinded to the clinical diagnosis reviewed the images. In case of a disagreement, a third observer served as an adjudicator. The graders assessed neuroretinal rim thinning (localized or diffuse), peripapillary RNFL defects (localized or diffuse), vertical and horizontal C/D ratios, and peripapillary atrophy. The vertical and horizontal C/D ratios were measured in the vertical and horizontal meridians respectively, considering the longest C/D diameter, which could be at oblique axis, not necessarily at 90 and 180-degree.

The eyes underwent imaging with the CSLO HRT II (software version 3.1.2; Heidelberg Engineering GmbH, Heidelberg, Germany). Images were taken of undilated pupils by an experienced examiner who marked the disc margin on all eyes. All images were reviewed for proper optic disc centering, focus, and illumination; and the mean topography images had a standard deviation of < 35 µm. The parameters evaluated in this study were: *C/D area ratio* (global), *vertical* and *horizontal C/D ratio*, *cup shape measure*, and *rim areas* (global and sectors).

The eyes were also imaged by SLP GDx with variable corneal compensation (VCC) (software version 5.3.3; Carl Zeiss Meditec, Dublin, CA, USA). The exam was performed for patients with undilated pupils by an experienced examiner. Images with good quality required focused and evenly illuminated reflectance image with a centered optic disc, no atypical retardation image pattern, and quality score > 7. *Temporal-superior-nasal-inferior-temporal* (*TSNIT*) *average*, *superior average*, *inferior average*, and *nerve fiber indicator* (NFI) were the evaluated parameters.

TD-OCT imaging was performed with a Stratus OCT (software version 5.0.1; Carl Zeiss Meditec, Dublin, CA, USA) to measure the RNFL thickness and the optic disc topography in all eyes with dilated pupils. The scans were manually centered on the optic disc by an experienced examiner. The ONH measurements were obtained by the Fast Optic Disc scan protocol. Six radial scans in a spoke like pattern are centered on the disc. Each radial scan includes 128 measuring points. The machine determined automatically the disc margin. The RNFL thickness measurements were assessed using the Fast RNFL thickness protocol. Three scans, each composed of 256 A-scans, were automatically acquired consecutively using a 3.46-mm-diameter circle around the optic disc. The OCT software automatically created a mean image. Only scans with good quality were considered in this study, including those with signal strength > 7 and no misalignment or movement artifacts.[27, 28] The analyzed parameters included *rim area*, *C/D area ratio*, *vertical* and *horizontal C/D ratio*, as well as *average*, *inferior*, *nasal*, *superior*, and *temporal RNFL thicknesses*.

SD-OCT imaging was performed with a Spectralis OCT (software version 5.1.3; Heidelberg Engineering, Heidelberg, Germany) on patients with dilated pupils by an experienced examiner, to measure RNFL thickness. The examiner manually placed the scan around the optic disc. A total of 1536 A-scan points were acquired from a 3.45-mm circle centered on the disc. To increase the image quality, the device included an automatic real-time function that gathered multiple frames, and images were averaged to reduce noise. All images were reviewed to ensure that the scan was centered, with signal strength > 15 dB, and that there were accurate segmentation and no artifacts.[29] *Global*, *inferior*, *nasal*, *superior*, and *temporal RNFL thicknesses* were the evaluated parameters.

### Statistical analysis

Only one eye per patient was included in the analysis. If both eyes of the patient were eligible, one eye was randomly selected. Data were analyzed using Stata software version 13.1 (StataCorp LP, College Station, Texas, USA) and PASW Statistics 18 (IBM, Armonk, New York, USA). Mean, standard deviation (SD), median, first (Q1) and third quartiles (Q3), as well as absolute and relative frequencies were used for descriptive analysis. The independent Student’s t test was used to compare age, IOP, and optic disc measurements between healthy and glaucoma groups. The Mann–Whitney test was used to compare visual acuity, SAP MD index, and DDLS between healthy and glaucoma groups. The Fisher’s exact test was used to compare race and gender between healthy and glaucoma groups.

DDLS sensitivity and specificity with 95% confidence intervals (CI) were calculated, in which a DDLS score > 5 was considered indicative of glaucomatous damage (Fig 1). Nonparametric receiver operating characteristic (ROC) curves were built and the area under the ROC curve (AROC) was calculated. Pair-wise comparison of AROC were performed between DDLS scores and structural and functional parameters.

The correlation between the DDLS score and structural and functional measurements was analyzed using Spearman’s rank correlation test. The significance level was set at 0.05.

## Results

One hundred and one eyes of 101 participants were enrolled in this study. Fifty-four eyes were healthy (53%), and 47 eyes had POAG (47%), including 16 preperimetric glaucoma (34%) cases. The glaucoma group was significantly older, with relatively worse visual acuity and higher IOP, C/D ratios, SAP MD indices, and DDLS scores (Table 1). Glaucoma group had SAP MD average (SD) of −5.48 (6.81) dB and the normal group of −1.12 (1.18) dB.

**Table 1:** Demographic and clinical ocular characteristics of the groups.

		Group	
Variable	Normal	Glaucoma	*P*
Number of patients (%)	54 (53%)	47 (47%)	N.A.
Age (year), mean (SD)	58.2 (9.1)	65.0 (10.2)	< 0.001
Race, n (%)			0.58
White	24 (44)	19 (40)	
Black	6 (11)	6 (13)	
Asian	2 (3.7)	5 (11)	
Mixed	22 (41)	17 (36)	
Gender, n (%)			0.69
Male	22 (41)	17 (36)	
Female	32 (59)	30 (64)	
IOP (mmHg), mean (SD)	13.7 (3.4)	15.9 (3.4)	0.002
Visual acuity (Decimal), median (Q1 to Q3)	1.0 (0.8 to 1.0)	0.7 (0.5 to 1.0)	< 0.001
Vertical optic disc diameter (mm), mean (SD)	1.81 (0.21)	1.79 (0.21)	0.54
Cup-to-disc ratio			
Vertical, mean (SD)	0.42 (0.19)	0.77 (0.12)	< 0.001
Horizontal, mean (SD)	0.38 (0.19)	0.71 (0.14)	< 0.001
Visual field MD index (dB), median (Q1 to Q3)	–1.09 (–1.73 to –0.25)	–2.95 (–5.83 to –1.08)	< 0.001
DDLS score, median (Q1 to Q3)	3 (3 to 4)	5 (4 to 7)	< 0.001

Abbreviations: N.A., not applicable; SD, standard deviation; n = number; IOP, intraocular pressure; Q1, first quartile; Q3, third quartile; MD, mean deviation; DDLS, Disc Damage Likelihood Scale.

Most of the optic discs (85%) had average size (from 1.5 to 2.0 mm), 3% were small and 12% were large (Fig 1). Forty-one eyes (41%) had DDLS > 5, of which sixteen (16%) eyes had DDLS > 7 and three (3%) had DDLS = 10.

Data from some tests were not analyzed due to inadequacy in image quality: Spectralis OCT (three eyes), Stratus OCT RNFL thickness (two eyes), Stratus OCT ONH (10 eyes), HRT II (13 eyes), and GDx-VCC (16 eyes).

A DDLS score > 5 (indicative of ONH glaucomatous damage) had a sensitivity of 74% (95% CI 60%‒86%) and specificity of 88% (95% CI 77%‒96%).

**Fig 2:**
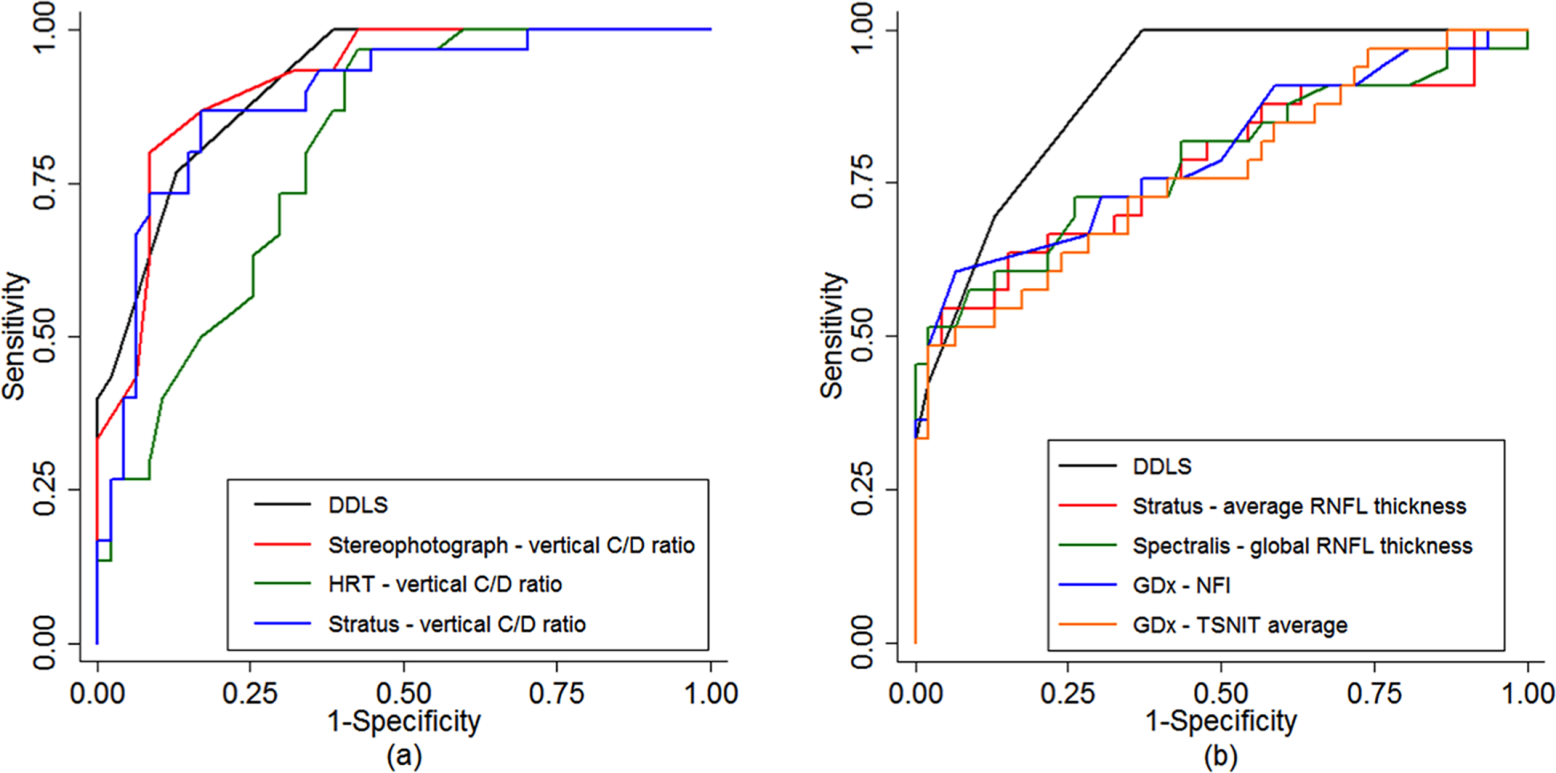
**ROC curves of DDLS score and of optic disc (a) and RNFL (b) best parameters of each diagnostic tool.** ROC, receiver operating characteristics; DDLS, Disc Damage Likelihood Scale; RNFL, retinal nerve fiber layer; C/D, cup-to-disc; HRT, Heidelberg retina tomograph; GDx, scanning laser polarimetry; NFI, nerve fiber indicator; TSNIT, temporal-superior-nasal-inferior-temporal.

The AROCs of the selected parameters are shown in Table 2. The DDLS (AROCs 0.92) and the vertical and horizontal C/D ratio (AROC 0.94 and 0.91, respectively) had excellent accuracies to discriminate glaucomatous from healthy eyes, with no statistically significant differences among them. The best ONH parameters were more accurate (AROC 0.83–0.94) than the best RNFL parameters of each diagnostic tool (0.79–0.83), followed by the SAP MD index (0.74). The only exception was the Spectralis OCT *global RNFL thickness*, which had the same accuracy as the HRT II *vertical C/D ratio* (0.83) [Table 2 and Fig 2(A) and 2(B)]. The DDLS diagnostic accuracy was better than the accuracy of the HRT II parameters, the Stratus and Spectralis OCT RNFL thicknesses, and the SAP MD index. There was no statistically significant differences between the accuracies of the DDLS and the OCT Stratus *vertical* (0.89) and *horizontal* (0.86) *C/D ratios*, OCT Stratus *C/D area ratio* (0.89), and GDx-VCC *NFI* (0.81) (Table 2).

**Table 2:** Areas under the receiver operating characteristic curve (AROC) of the diagnostic tools.

Exam		AROC	(95% CI)	P*	*P* ^†^
**DDLS**		0.92	(0.87–0.97)	<0.001	N.A.
**Disc stereophotograph**					
Vertical C/D ratio		0.94	(0.89–0.98)	<0.001	0.51
Horizontal C/D ratio		0.91	(0.85–0.96)	<0.001	0.66
**HRT II**					
C/D area ratio (global)		0.81	(0.72–0.90)	<0.001	0.006
Vertical C/D ratio		0.83	(0.74–0.91)	<0.001	0.01
Horizontal C/D ratio		0.79	(0.69–0.89)	<0.001	0.006
Cup shape measure		0.77	(0.66–0.87)	<0.001	0.002
Rim Area					
Global		0.70	(0.58–0.82)	0.002	<0.001
Temporal		0.66	(0.54–0.79)	0.01	<0.001
Superior Temporal		0.75	(0.64–0.86)	<0.001	<0.001
Inferior Temporal		0.70	(0.58–0.82)	0.002	<0.001
Nasal		0.62	(0.50–0.74)	0.07	<0.001
Superior Nasal		0.70	(0.57–0.82)	0.002	<0.001
Inferior Nasal		0.63	(0.50–0.75)	0.05	<0.001
**Stratus OCT**					
Optic disc					
Rim area		0.82	(0.73–0.91)	<0.001	0.03
C/D area ratio		0.89	(0.82–0.96)	<0.001	0.44
Vertical C/D ratio		0.89	(0.82–0.96)	<0.001	0.46
Horizontal C/D ratio		0.86	(0.79–0.94)	<0.001	0.19
RNFL thickness					
Average		0.79	(0.69–0.89)	<0.001	0.01
Inferior		0.77	(0.67–0.87)	<0.001	0.007
Nasal		0.73	(0.62–0.84)	<0.001	0.004
Superior		0.71	(0.60–0.83)	0.001	0.001
Temporal		0.61	(0.50–0.73)	0.07	<0.001
**Spectralis OCT**					
RNFL thickness					
Global		0.83	(0.75–0.91)	<0.001	0.04
Inferior		0.81	(0.72–0.90)	<0.001	0.01
Nasal		0.75	(0.65–0.85)	<0.001	0.003
Superior		0.80	(0.71–0.89)	<0.001	0.01
Temporal		0.69	(0.59–0.80)	0.001	<0.001
**GDx-VCC**					
NFI		0.81	(0.72–0.91)	<0.001	0.06
TSNIT Average		0.78	(0.68–0.89)	<0.001	0.02
Superior Average		0.77	(0.66–0.88)	<0.001	0.01
Inferior Average		0.76	(0.65–0.87)	<0.001	0.007
**Visual field mean deviation index**		0.74	(0.62–0.85)	<0.001	0.001

Abbreviations: CI, confidence interval; DDLS, Disc Damage Likelihood Scale; N.A., not applicable; C/D, cup-to-disc; HRT, Heidelberg retina tomograph

OCT, optical coherence tomography; RNFL, retinal nerve fiber layer; GDx-VCC, scanning laser polarimetry with variable corneal compensation; NFI, nerve fiber indicator; TSNIT, temporal-superior-nasal-inferior-temporal.

* Comparison with AROC of 0.05

^†^ Comparison with AROC for DDLS.

The correlations between DDLS score and C/D ratio, SAP MD index, OCT (Stratus and Spectralis), GDx-VCC, and HRT II parameters are described in Table 3. The DDLS system had significant moderate to strong (*r =* −0.38 to −0.79) correlations with all evaluated structural and functional parameters, except for OCT Stratus *temporal RNFL thickness (r =* −0.24). We found a moderately negative correlation between DDLS and SAP MD (*r* = −0.47, Table 3).

**Table 3:** Correlation between DDLS score and cup-to-disc ratio, visual field mean deviation, OCT, GDx-VCC and HRT parameters.

Exam	*r*	*P*
**Disc stereophotograph**		
Vertical C/D ratio	0.79	< 0.001
Horizontal C/D ratio	0.74	< 0.001
**HRT II**		
C/D area ratio (global)	0.77	< 0.001
Vertical C/D ratio	0.77	< 0.001
Horizontal C/D ratio	0.66	< 0.001
Cup shape measure	0.59	< 0.001
Rim Area		
Global	− 0.61	< 0.001
Temporal	− 0.56	< 0.001
Superior Temporal	− 0.62	< 0.001
Inferior Temporal	− 0.67	< 0.001
Nasal	− 0.38	< 0.001
Superior Nasal	− 0.52	< 0.001
Inferior Nasal	− 0.47	< 0.001
**Stratus OCT**		
Optic disc		
Rim area	− 0.64	< 0.001
C/D area ratio	0.72	< 0.001
Vertical C/D ratio	0.75	< 0.001
Horizontal C/D ratio	0.63	< 0.001
RNFL thickness		
Average	− 0.61	< 0.001
Inferior	− 0.61	< 0.001
Nasal	− 0.39	< 0.001
Superior	− 0.52	< 0.001
Temporal	− 0.24	0.02
**Spectralis OCT**		
RNFL thickness		
Global	− 0.64	< 0.001
Inferior	− 0.61	< 0.001
Nasal	− 0.42	< 0.001
Superior	− 0.62	< 0.001
Temporal	− 0.41	< 0.001
**GDx-VCC**		
NFI	0.51	< 0.001
TSNIT Average	− 0.50	< 0.001
Superior Average	− 0.50	< 0.001
Inferior Average	− 0.45	< 0.001
**Visual field mean deviation index**	− 0.47	< 0.001

Abbreviations: DDLS, Disc Damage Likelihood Scale; OCT, optical coherence tomography; GDx-VCC, scanning laser polarimetry with variable corneal compensation; HRT, Heidelberg retina tomograph; r, Spearman's rank correlation coefficient; C/D, cup-to-disc; RNFL, retinal nerve fiber layer; NFI, nerve fiber indicator; TSNIT, temporal-superior-nasal-inferior-temporal.

DDLS had the best correlation with vertical C/D ratio (*r* = 0.79). DDLS had also strong correlation with horizontal C/D ratio (0.74); HRT *vertical C/D ratio* (0.77) and *global C/D area ratio* (0.77); and with Stratus OCT *vertical C/D ratio* (0.75) and *C/D area ratio* (0.72) (Table 3).

**Fig 3:**
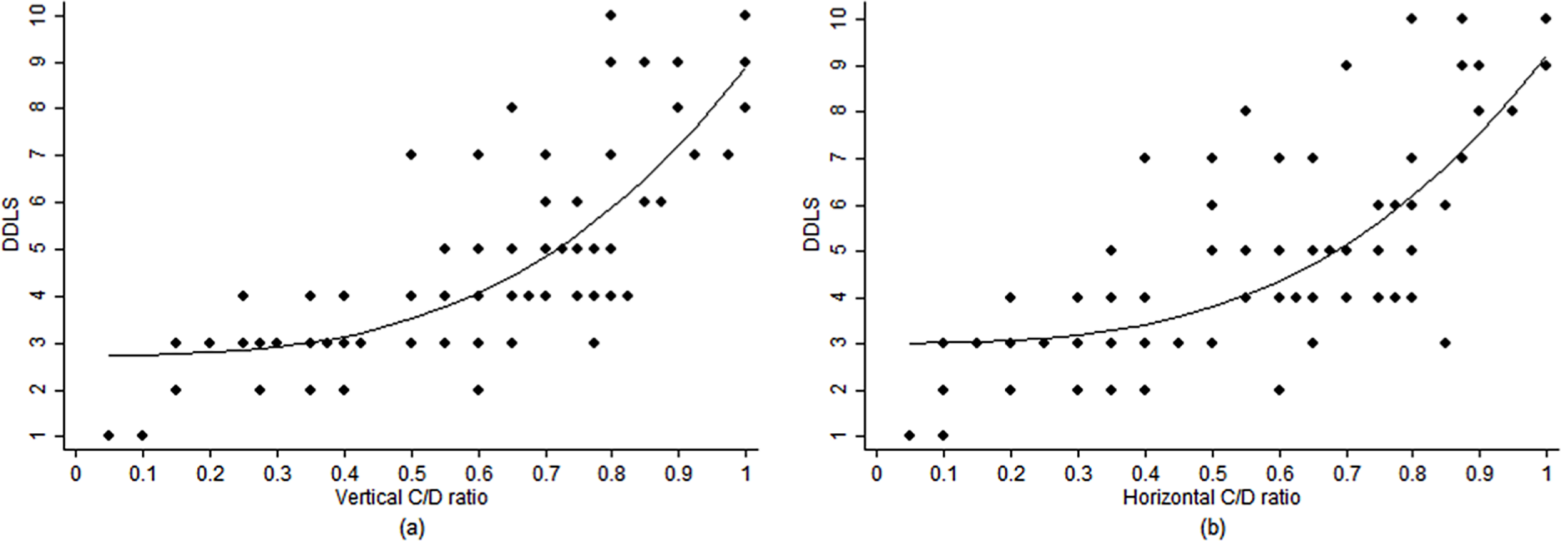
Scatterplot showing the association between DDLS and C/D ratio. (a) DDLS and vertical C/D ratio, (b) DDLS and horizontal C/D ratio. Abbreviations: DDLS, Disc Damage Likelihood Scale; C/D, cup-to-disc.

There was a monotonic non-linear correlation between DDLS and C/D ratio, with a greater increase of DDLS stages in the presence of larger vertical or horizontal C/D ratios (Fig 3).

The correlations between DDLS and *rim areas* from HRT II were all moderate (*r =* −0.38 to −0.67); the strongest correlation was with *inferior temporal rim area* and the worst correlations were with *nasal and inferior nasal rim areas* (Table 3).

The higher DDLS correlation with Stratus RNFL thickness was with *average* and *inferior RNFL thicknesses* (both *r* = −0.61); and the weaker correlations were with *temporal* and *nasal areas* (*r* = −0.24 and −0.39, respectively, Table 3).

The Spectralis RNFL thickness had moderately negative correlations with DDLS. The strongest DDLS correlation was with *global RNFL thickness* (*r* = −0.64), followed by *superior* and *inferior thicknesses* (*r* = –0.62 and −0.61, respectively); the lowest DDLS correlation was with *temporal* and *nasal areas* (*r* = –0.41 and −0.42, respectively, Table 3).

The correlations between DDLS and the parameters acquired by GDx-VCC were moderately negative: *TSNIT average* (*r* = −0.50); *superior average* (*r* = −0.50); and *inferior average* (*r* = −0.45), except for the correlation with the *NFI* parameter, which was moderately positive (*r* = 0.51, Table 3).

## Discussion

We analyzed the DDLS diagnostic accuracy and its correlations with C/D ratio; SAP MD; TD- and SD-OCT and GDx-VCC RNFL thicknesses; and with TD-OCT and HRT II ONH parameters. The ONH clinical evaluation with DDLS and C/D ratio demonstrated excellent accuracy in distinguishing glaucomatous from healthy eyes. Moreover, the DDLS had significant moderate to strong correlations with most structural and functional measurements. To our knowledge, this is the first study comparing the DDLS accuracy and evaluating this system correlation with Spectralis OCT and GDx-VCC.

Several other automated devices can be used to objectively evaluate the optic disc; however, clinical examination of the optic disc is still one of the most important steps when evaluating glaucomatous patients. One cannot rely only on ONH and RNFL imaging devices for glaucoma diagnosis because they are expensive, the technology is always evolving, and information obtained by different techniques is not interchangeable.[8, 30–33] This can limit their availability, as well as their usefulness, when applied to patients with common, widespread chronic illnesses, such as glaucoma.[8] The DDLS, a quantitative optic disc staging system which takes into consideration disc size and position of the rim thinning, through an inexpensive method and by using the readily available slit lamp, can be used in different clinical settings, including areas with different levels of development and where resources are scarce. The DDLS permits grading optic discs reliably into different clinically stages.[11] Our findings suggest that the DDLS system is useful to detect glaucomatous damage, and has a diagnostic accuracy similar to newer and more expensive imaging devices.

Most of the studies that analyzed the DDLS diagnostic accuracy and correlations with other functional and structural tests calculated the DDLS stage through slit lamp.[10, 13, 14, 17–19] When comparing the optic disc evaluation using the DDLS assessed by slit lamp or by stereophotographs, the major advantage of the DDLS through slit lamp is its lower cost. Stereophotographs require a fundus camera, which is more expensive [33] and less likely to be as available and as simple to maintain as a slit lamp. The main limitation of the DDLS determined by stereophotographs is the absence of a reliable quantitative method for estimating disc size.[7, 12] Additionally, stereophotographs can present bad image and stereo quality. Therefore, we calculated the DDLS using a slit lamp. Nonetheless, DDLS performed by stereophotographs also has advantages: it is more confortable for the patient, as it is less time-consuming, and it gives the examiner a greater period of time to analyze the disc with no eye movements or blinking. Moreover, stereophotographs are very useful, especially for glaucoma progression detection, but this was not evaluated herein.

In this report, a DDLS score > 5, as indicative of ONH glaucomatous damage, had moderate sensitivity and good specificity, probably because most of the glaucoma cases were mild (average SAP MD = −5.48 dB), and 34% of the glaucoma patients had preperimetric disease. Also, the ONH clinical evaluation using DDLS to discriminate POAG from healthy eyes, as well as vertical and horizontal C/D ratios, had excellent diagnostic accuracy. This result could be in part overestimated since the adopted diagnosis criteria were based on the clinical ONH and RNFL structural exam. Previous studies also reported large AROCs (0.91–0.94) for DDLS, but had worse results than ours for vertical and horizontal C/D ratios (0.80–0.84 and 0.76, respectively).[14, 18, 20]

In our study, the similar accuracies for DDLS (AROC 0.92) and for vertical and horizontal C/D ratios (0.94 and 0.91, respectively) may be likely explained for 3 reasons: 1) Vertical and horizontal C/D ratios were estimated according to the longest C/D diameter, not necessarily at 90 and 180-degree axis, respectively; consequently the narrowest width of the neuroretinal rim, in the vertical and horizontal meridians, were measured; 2) There was a monotonic non-linear correlation between DDLS and C/D ratios (vertical and horizontal), indicating more advantages of DDLS system in the presence of optic discs with larger C/D ratios. The DDLS nomogram presents stages > 7 for optic discs with at least 45-degree of rim absence for any disc size; only 16% of the eyes herein were graded in such more advanced stages, in which DDLS could have better accuracy than C/D ratio. For discs with DDLS > 7, all the circumferential extent of rim absence can still be evaluated up to stage 10, while the C/D ratio has only a small amount to be enlarged (Fig 3); and 3) Most of optic discs had average size, cases where the C/D ratio is usually more valuable. Larger discs (with usually larger cups) can be assumed to be glaucomatous, whereas smaller discs (with consequent smaller cups) can be misinterpreted as healthy.[6, 8]

The vertical and horizontal C/D ratios were strongly correlated to DDLS, corroborating with the results of previous studies (C/D ratio vertical 0.73−0.86; horizontal 0.79−0.80).[18, 20] The DDLS also presented strong correlation with HRT II *vertical C/D ratio* (0.77) and *C/D area ratio* (0.77), as well as with the Stratus OCT *vertical C/D ratio* (0.75) and *C/D area ratio* (0.72). Since the DDLS is based on the width of the neuroretinal rim or the circumferential extent of rim absence, its significant positive correlation with the C/D ratios is an expected result.

The SAP MD correlation with DDLS and diagnostic accuracy was moderate, and other reports showed DDLS moderate-to-strong correlations with SAP MD (−0.55 to −0.79).[10, 13, 14, 16, 18–20] The SAP MD weaker correlation and performance reported herein can be explained by our adopted glaucoma diagnosis criteria, based on the presence of structural damage regardless visual field results, which allowed the preperimetric glaucoma inclusion.[34] Also, most of the glaucomatous eyes in the present study were mild; generally, in early stage glaucoma, changes in ONH precede visual field defects.[35]

HRT II parameters have already been demonstrated as good indicators of structural ONH damage.[25] We found moderate-to-strong correlations between DDLS and all evaluated HRT II parameters. Previous reports also demonstrated a significant correlation between DDLS and the various parameters acquired by HRT II.[13, 16] Considering the *rim area* parameters, the current study showed that *inferior temporal rim area* had the strongest correlation with the DDLS, and the *nasal area* had the weakest. These results are in agreement with Danesh-Meyer et al.,[13] which demonstrated that the DDLS had the highest correlation with the *inferior temporal rim area* and the lowest correlation with the *nasal rim area* measurement from HRT. A possible explanation is that in our sample most of the cases had mild glaucoma; usually glaucomatous neuroretinal rim loss begins at the inferotemporal region and then progresses following the inferior-superior-nasal-temporal pattern.[36]

We found moderate-to-strong correlations between DDLS and all evaluated Stratus OCT ONH parameters. The strongest DDLS correlations were with *vertical C/D ratio* (*r* = 0.75) and *C/D area ratio* (0.72), followed by *rim area* (−0.64) and *horizontal C/D ratio* (0.63). Abdul Majid et al.[18] also reported Stratus OCT *vertical C/D ratio* (0.59) and *C/D area ratio* (0.66) as one of the best parameters correlated to DDLS, but with moderate correlation. Han et al.[20] demonstrated strong correlations between DDLS and Cirrus OCT *rim area* (−0.75) and *vertical C/D ratio* (0.74).

In the present study, the DDLS showed moderately negative correlations with RNFL thickness evaluation by Stratus and Spectralis OCT, but not with the Stratus OCT *temporal area* which had weak correlation. The strongest DDLS correlations occurred with the *average* and *inferior RNFL thickness* parameters for Stratus OCT and the *global* and *superior/inferior RNFL thickness* for Spectralis OCT. For both OCT devices, the weakest correlations were with *temporal* and *nasal areas*, corroborating Abdul Majid et al.’s report, which presented the following correlations for Stratus OCT: *inferior* (−0.62), *average* (−0.62), *superior* (−0.60), *temporal* (−0.37) and *nasal* (−0.38) *RNFL thickness*.[18] Other reports evaluated the DDLS correlation only with the *average RNFL thickness* by Stratus (−0.85) and Cirrus (−0.70) OCT RNFL parameters and found a stronger correlation than we did herein.[19, 20]

The correlations between DDLS and the parameters obtained by GDx-VCC were significantly moderate negative, except for the *NFI* parameter that had a positive moderate correlation. Also, *NFI* had the best accuracy to discriminate glaucomatous from healthy eyes. We did not find any published reports in which DDLS and GDx-VCC have been correlated.

Our study main limitation was the fact that, to include eyes with preperimetric glaucoma, the adopted glaucoma diagnosis criteria were based in the presence of ONH and RNFL structural damage, which could overestimate the structural parameters results.[34] In a DDLS correlation study that classified glaucoma based on ONH and SAP damage, Abdul Majid et al.[18] attempted to minimize selection bias by carrying out a separate subset analysis, in which the ONH rim thinning criteria was excluded from the optic disc evaluation, since the ONH assessment, especially the neuroretinal rim, is part of the DDLS system, and could result in an overestimation of the DDLS correlations. However, there were no significant changes in the correlations between the DDLS and the studied parameters (C/D ratio, Stratus OCT parameters and SAP MD index).[18] Another limitation of our study was that all exams were obtained within a 6-month period, although, the expected glaucoma progression in this short period of time in POAG patients with intraocular pressure under the target level on hypotensive medications is unlikely to significantly occur and influence our results.

In conclusion, the optic disc clinical evaluation with the DDLS system and the C/D ratio demonstrated excellent accuracy in distinguishing glaucomatous from healthy eyes. The DDLS had significant moderate to strong correlations with most of the structural and functional parameters. These findings stress the importance of optic disc clinical examination to detect glaucoma in a clinical scenario.

## Supporting information

S1 File Raw data. (XLS)
